# Can knowledgeable experts assess costs and outcomes as if they were ignorant? An experiment within precision medicine evaluation

**DOI:** 10.1017/S0266462323002714

**Published:** 2023-11-17

**Authors:** Thamonwan Dulsamphan, Parntip Juntama, Chotika Suwanpanich, Wanrudee Isaranuwatchai, Madison Silzle, Sathida Poonmaksatit, Ponghatai Boonsimma, Vorasuk Shotelersuk, Anannit Visudtibhan, Apasri Lusawat, Wuttichart Kamolvisit, Nattiya Kapol, Surasit Lochid-amnuay, Namfon Sribundit, Nathapol Samprasit, Alec Morton, Yot Teerawattananon

**Affiliations:** 1 Health Intervention and Technology Assessment Program, Ministry of Public Health, Nonthaburi, Thailand; 2 Division of Neurology, Department of Pediatrics, Faculty of Medicine, Chulalongkorn University, Bangkok, Thailand; 3 Center of Excellence for Medical Genomics, Medical Genomics Cluster, Department of Pediatrics, Faculty of Medicine, Chulalongkorn University, Bangkok, Thailand; 4 Excellence Center for Genomics and Precision Medicine, King Chulalongkorn Memorial Hospital, The Thai Red Cross Society, Bangkok, Thailand; 5 Division of Neurology, Department of Pediatrics, Faculty of Medicine, Ramathibodi Hospital, Mahidol University, Bangkok, Thailand; 6 Neurological Institute of Thailand, Bangkok, Thailand; 7 Department of Health Consumer Protection and Pharmacy Administration, Faculty of Pharmacy, Silpakorn University, Nakhon Pathom, Thailand; 8 Bangpakok 8 Hospital, Bangpakok Hospital Group, Bangkok, Thailand; 9 Department of Management Science, Strathclyde Business School, University of Strathclyde, Glasgow, UK

**Keywords:** precision medicine, judgment, healthcare costs, exome sequencing, hindsight bias

## Abstract

**Objectives:**

The purpose of this study is to evaluate the validity of the standard approach in expert judgment for evaluating precision medicines, in which experts are required to estimate outcomes as if they did not have access to diagnostic information, whereas in fact, they do.

**Methods:**

Fourteen clinicians participated in an expert judgment task to estimate the cost and medical outcomes of the use of exome sequencing in pediatric patients with intractable epilepsy in Thailand. Experts were randomly assigned to either an “unblind” or “blind” group; the former was provided with the exome sequencing results for each patient case prior to the judgment task, whereas the latter was not provided with the exome sequencing results. Both groups were asked to estimate the outcomes for the counterfactual scenario, in which patients had not been tested by exome sequencing.

**Results:**

Our study did not show significant results, possibly due to the small sample size of both participants and case studies.

**Conclusions:**

A comparison of the unblind and blind approach did not show conclusive evidence that there is a difference in outcomes. However, until further evidence suggests otherwise, we recommend the blind approach as preferable when using expert judgment to evaluate precision medicines because this approach is more representative of the counterfactual scenario than the unblind approach.

## Introduction

Economic evaluation of precision medicine technologies is particularly important for healthcare decision-making, as precision medicine tends to have high costs and niche markets (1–3). To be included in public reimbursement programs, these technologies must undergo various assessments to demonstrate their values, such as clinical benefit or cost effectiveness (1–3). These assessments require a comparison between the costs and outcomes of a precision medicine intervention and a comparator (4).

While a randomized control trial is widely considered to be the preferred method to compare the costs and outcomes of a new intervention (5), it is rarely feasible, and, sometimes, unethical to do so for precision medicines. Alternatively, other common methods such as observational studies are often limited by the lack of control groups or small cohorts. Given this limited availability of data, it is useful to employ simulative methods to estimate the cost effectiveness of precision medicines, such as through modeling and expert judgment (6–11). These methods allow for hypothetical data to be used as a comparator where no real-world data are available.

Expert judgment is an important approach for the economic evaluation of precision medicines that sometimes requires experts to judge counterfactual scenarios, defined as “thinking about what did not happen but could have happened.” (12) Often, such as for emerging technologies, experts predict outcomes from the use of precision medicine for a cohort of patients who received standard of care (9;13–15). Alternatively, experts may be asked to predict the outcomes from the standard of care when patients, in fact, received a precision medicine test or treatment (16–18). It is expected that estimated outcomes may be more accurate in the latter, rather than the former, case, given that experts usually have more experience with the standard of care. Thus, experts are able to use this knowledge base to better inform their predictions of the counterfactual scenario.

However, there is an additional conceptual and psychological difficulty when experts are asked to estimate the standard-of-care outcomes for patients who have received a precision medicine test, genetic sequencing, for example. Experts who know the results of a test may find that this knowledge influences the answers they provide when asked to consider patient outcomes without the use of precision medicines. Psychology research suggests this type of retrospective thinking is subject to hindsight bias, which is defined as the “belief that an event is more predictable after it becomes known than it was before it became known.” (19) Individuals have a difficult time recreating or achieving the feeling of uncertainty they would have if outcomes were unknown (19).

This would have consequences for the judgments provided by “unblind” experts. In this method, experts are asked to estimate the outcomes for the counterfactual scenario(s) as if they did not know the results of the test or treatment, thus allowing the comparison of multiple possible costs and health outcomes for the same cohort of patients. This method is common, and sometimes unavoidable, given that experts are often familiar with the patient populations they are asked to judge (16–18). However, due to hindsight bias, unblind experts would be less likely to consider the possibility of alternative outcomes and assign higher probabilities to a correct or real-world outcome than if they had not known the real-world outcome prior to the judgment task. Such bias could result in inaccurate outcome estimations of the counterfactual scenario and subsequent undervaluation of the intervention for consideration in healthcare decision-making processes.

This study employed a blind approach, in which experts would not be provided with outcomes prior to a judgment task. The study objective is to compare the costs and health outcomes between the unblind and blind approaches in estimating the counterfactual scenario using expert judgment. The precision medicine technology evaluated was exome sequencing (ES) used for pediatric patients with intractable epilepsy in Thailand. The primary outcome evaluated was direct medical cost, and secondary outcomes were the accuracy of the final diagnosis and the accuracy of predicting prognosis.

We hypothesized that these two approaches would result in different outcomes due to the effect of hindsight bias in the unblind experts. We predicted that unblind experts would estimate greater diagnostic and prognostic accuracy than blind experts. We also predicted that the estimated direct medical costs would be much lower among unblind experts because they would not investigate alternative diagnoses as much as blind experts who would experience greater uncertainty.

## Methods

### Study design

An experimental study was set up using clinical experts randomly assigned to an unblind or blind group to provide expert judgments on three real-world patient cases. Experts were asked to investigate and provide likely treatments and potential diagnoses for a total of three visits in each of the three cases. At the third visit in each case, they were also asked to predict the prognosis of the patient two years post-visit.

In this study, the unblind group was informed of the final molecular diagnoses by ES at the beginning of the exercise for each patient case but asked to perform the judgment task as if they did not know the molecular diagnoses. The blind group was asked to perform the judgment task without knowing the final molecular diagnoses for each patient case.

### Sample selection and sampling

Experts were recruited from the Child Neurology Association, Thailand, according to the following criteria 1) currently practicing physicians specializing in pediatric neurology and 2) willing to participate in the workshop in person on January 21, 2022, in central Bangkok. A total of fourteen experts were included in the judgment task to maintain a minimum of five experts per group, as recommended by the Reference Case for Health Technology Assessment in Healthcare Decision-Making (20). Each expert was compensated 2,000 Thai Baht for participation and travel costs.

Experts were randomly assigned to the unblind or the blind group. Experts were stratified by whether they had more or less than five years of experience, whether they worked in Bangkok and metropolitan areas or other provinces, and whether they worked in public or private hospitals.

### Patient case selection

Patient cases were selected by stratified random sampling from a cohort of 104 intractable epileptic pediatric patients who were treated at Chulalongkorn Memorial Hospital between June 2016 and December 2020 and underwent testing by ES (21). Patients within this cohort were first classified based on the outcome of ES – whether a definite molecular diagnosis was made or not – and whether the subsequent treatment plan changed as a result. Strata were defined as 1) no definite diagnosis and treatment plan did not change, 2) definite diagnosis and treatment plan did not change, and 3) definite diagnosis and treatment plan changed. One case from each group was randomly selected for use in the judgment task, such that cases 1, 2, and 3 correlate to strata 1, 2, and 3, respectively (Table 1).

**Table 1.** Summary of direct medical cost analysis (linear regression and bootstrap results) between unblind and blind groups by patient case

		Linear regression (*N* = 14 in each case)		
		Cost of investigation	Cost of treatment	Total cost
	Case type	ΔC (95% CI)	ΔC (95% CI)	ΔC (95% CI)
Case 1	No definite diagnoses by ES & no change in treatment plan	7,946 (−13,207 to 29,098)	−1,024 (−34,093 to 32,045)	6,922 (−31,370 to 45,213)
Case 2	Definite diagnosis by ES & no change in treatment plan	7,449 **(−**10,545 to 25,443.4)	−31,106 (−80,364 to 18,152)	−23,657 (−77,021 to 29,707)
Case 3	Definite diagnosis by ES & subsequent change in treatment plan	7,574 (−11,094 to 26,242)	−451 (−13,097 to 12,196)	7,123 (−19,313 to 33,559)
		Bootstrap of regression results (*N* = 14 in each case)		
		Cost of investigation 95% CI	Cost of treatment 95% CI	Total cost 95% CI
Case 1	No definite diagnoses by ES & no change in treatment plan	−9,714 to 25,606	−28,063 to 26,015	−24,546 to 38,390
Case 2	Definite diagnosis by ES & no change in treatment plan	−7,900 to 22,799	−72,238 to 10,026	−68,732 to 21,418
Case 3	Definite diagnosis by ES & no change in treatment plan	−8,202 to 23,349	−11,124 to 10,223	−15,276 to 29,522

*Note*: ΔC indicates the difference in mean direct medical cost between unblind and blind groups (ΔC = mean cost_blind_ – mean cost_unblind_).

### Materials and data collection

Development of the judgment task procedure closely followed recommendations from the Reference Case for Health Technology Assessment in Healthcare Decision-Making (14), with several key changes made for the purpose of this study. The materials used for data collection were adapted from forms used within hospital settings to maintain familiarity with clinicians and reduce procedure-based confusion and error and were provided in Thai language. The materials are available upon request.

The judgment task was piloted twice with two experts not recruited for the study. Although the pilots occurred virtually, the process was conducted in a way similar to that of the in-person workshop as possible. Feedback from the pilot helped inform the judgment protocols and several revisions were made to the data collection forms. For example, it was decided that all information provided to experts would reflect real-world information from each patient case. Thus, if an expert ordered an investigation that was not available from the real-world case at that visit, they were informed the test result was not available, but the investigation was still included for calculating cost outcomes. Additionally, if that test result became available at a later visit, experts who had previously ordered the test were informed of the result, as well as the real timing associated with the result. In conclusion, no artificial information was generated by the research team for this judgment task.

The workshop occurred in person, and the two groups were assigned to separate rooms. Throughout the judgment task, experts were not allowed to interact with other experts. This change was made from the Reference Case as this follows the traditional approach for this type of study (13;14;22) and more clearly exposes differences between groups and individuals.

Two clinical experts coordinated the task for each group. They were responsible for providing instructions, timing each task, and solving emerging problems. Twelve facilitators were used to manage individual experts by providing materials as needed throughout the task and answering individual questions. All coordinators and facilitators were trained for their respective roles. Finally, each expert was assigned a participant code so that the expert’s personal information was de-identified with their judgment task outcomes. All experts were briefed on the objectives, procedures, and expected benefits of this study and provided consent to participate prior to the workshop.

### Outcomes

**Figure 1.**
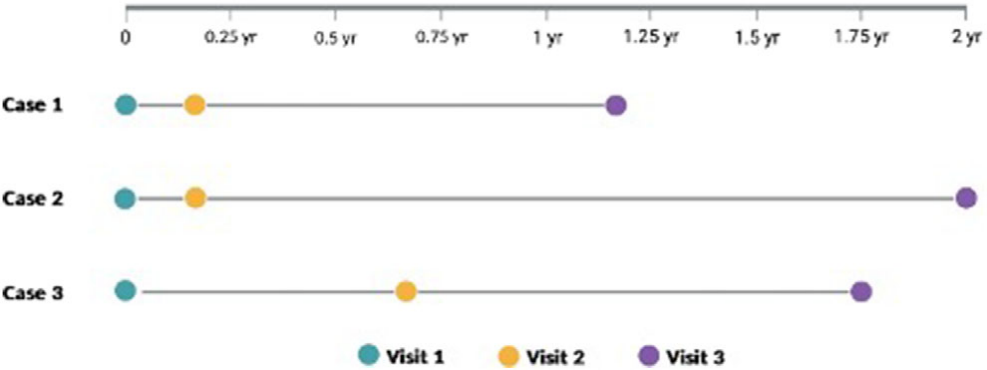
Real-world timing of patient visits 1, 2, and 3 for all three case studies.

The primary outcome was the total direct medical cost in 2022 Thai Baht (35 Thai Baht = 1 USD), which included both estimated costs from labs ordered during the investigation and treatments given for each patient case. Secondary outcomes were accuracy of the final diagnosis and accuracy of predicting prognosis, scored from 0 to 100. For each visit, experts ordered tests from a predetermined list and then wrote a treatment plan based on the results of the tests they ordered. Experts were then asked to provide up to three possible diagnoses for the patient with an associated confidence level such that the confidence of each diagnosis together totaled 100 percent. A score of 75 would refer to being 75 percent confident of the correct final diagnosis and a score of 100 would refer to being 100 percent confident of the correct final diagnosis. Experts repeated this process for visit two and visit three. At visit three, experts were also asked to provide a prognosis for the patient. Four prognosis options were given, comparing the frequency of seizures at the visit one to two years after the final visit: more frequent seizures, the same frequency of seizures, less frequent seizures, and no seizures. Experts were asked to provide a confidence level of each prognosis option such that the total confidence of each prognosis totaled 100 percent. Answers were scored based on the confidence level in the correct prognosis. For example, an expert who indicated 50 percent confidence in the correct prognosis would receive a score of 50. Experts repeated this process for all three cases. The timing of visits one, two, and three for each case varied depending on the real clinical practice and is depicted in Figure 1.

### Data analysis

Different statistical tests were used for the three outcomes. First, total direct medical cost was compared between the unblind and blind groups by each patient case using simple linear regression and a Mann–Whitney test. Given the small sample size of experts, the uncertainty of the findings was further characterized using a non-parametric approach of bootstrap at 1,000 times. Second, the accuracy of the final diagnosis between groups was compared using the Mann–Whitney test, whereas the comparison of accuracy of predicting prognosis between groups (the third outcome) employed a t-test based on this outcome’s distribution. Statistical significance was set at a p-value <0.05. All statistical analyses were performed using Stata version 16 (Stata, College Station, TX).

### Ethics approval

Ethical approval was received for this study from the Human Research Ethics Committee of Silpakorn University (COE 65.0120–006).

## Results

Of the 14 experts who participated in the workshop, most were affiliated with public hospitals (N = 12) located in Bangkok (N = 11). The average number of years of experience as a physician was 8.32 years, with the minimum experience being 1 year and the maximum experience being 27 years. Detailed information on the characteristics of the experts is listed in the Supplementary Table S1.

The comparison of direct medical costs between unblind and blind groups is summarized in Table 1 by the cost of investigation, cost of treatment, and total cost. The blind group had a greater mean cost of investigation but a lower mean cost of treatment than the unblind group for all three cases, though the differences were not significant by linear regression analysis. The mean total costs were higher in the blind group for Cases 1 and 3 but lower in Case 2 with no statistical significance. Results from bootstrap sampling showed no significant difference, however, with less uncertainty (i.e., smaller 95 percent confidence interval). Comparison of total cost between groups by each patient case by Mann–Whitney test resulted in the same findings.

Differences in the accuracy of the final diagnosis by patient case between the unblind and blind group were only seen in Case 2 (Table 2).

**Table 2.** Comparison of accuracy of final diagnosis between groups by Mann–Whitney U Test

Case	Group (*N* = 14)	Mean (SD)	*p*-value
1	Unblind	100 (0)	–
	Blind	100 (0)	
2	Unblind	94.3 (11.3)	.034
	Blind	47.1 (41.5)	
3	Unblind	99.9 (0.4)	–
	Blind	100 (0)	

No significant differences were seen between groups in average percent confidence in the correct predicted prognosis by t-test (Table 3).

**Table 3.** Comparison of percent confidence in correct predicted prognosis between groups by t-test

Case	Group (*N* = 14)	Mean % Confidence (SD)	95% CI
1	unblind	51.4 (21.9)	31.1, 71.7
	Blind	45.7 (15.1)	31.7, 59.7
2	unblind	22.1 (12.2)	10.9, 33.4
	Blind	19.3 (18.4)	2.3, 36.3
3	unblind	85.7 (12.7)	73.9, 97.5
	Blind	94.3 (7.9)	87.0, 101.6

*Note*: For Cases 1, 2, and 3, the correct prognoses were (a) more frequent seizures, (b) less frequent seizures, and (c) no seizures, respectively.

## Discussion

Differences in total direct medical costs for the counterfactual scenario between the unblind and blind approach, while not statistically significant, could have implications for decision-making. If the outcomes of either the unblind or blind approach in our study had been used to inform the decision to include ES in a universal coverage benefit package, they may have led to different conclusions. For example, an additional cost of 7,123 THB, as we see in the blind group for Case 3, is substantial enough to result in not funding ES for pediatric epilepsy cases in Thailand, whereas the unblind approach could result in funding.

Differences between costs and health outcomes between the unblind and blind groups can likely be explained by both effects of hindsight bias and differences in the type of cases presented. A higher cost of investigation was observed in the blind group for all three cases, which could be explained by greater uncertainty in diagnosis. More frequent changes in diagnoses between visits, as seen in the blind group in Cases 1 and 2 (Supplementary Table S2), likely indicate greater uncertainty and are accompanied by more investigation orders to compensate. This could also be due to the effects of overconfidence, a consequence of hindsight bias, in unblind experts (23). Unblind experts may overestimate their ability to diagnose a patient without molecular testing and overlook the possibility of alternative diagnoses when engaging in counterfactual thinking. For example, if an expert knows the outcome of ES is a diagnosis of KCNQ2-related epileptic encephalopathy, the expert may exaggerate their ability to treat this patient without ES, which hinders the ability to consider any alternative diagnoses when performing judgment tasks. Subsequently, unblind experts vary less in their diagnosis over time and request less investigation. Although this did not translate into significant differences in investigation costs, it is still a notable distinction that could be explored in further studies.

Despite the blind group also having higher investigation costs in Case 2, their total cost was lower than the unblind group due to considerably lower treatment costs. Differences in treatment costs are likely due to differences in the predicted diagnosis, as the diagnosis determines subsequent treatment plans. For example, the Case 2 disease type is very unlikely to be correctly diagnosed without ES, and it is expected that the unblind and blind groups had significant differences in diagnosis for this case and subsequent differences in treatment plans and costs. Likewise, the unblind and blind groups had minimal differences in treatment costs in Case 3 because all experts, regardless of blinding, correctly diagnosed this patient.

While the comparison of cost outcomes in our study did not show significant results, the direction of differences suggests that the unblind approach could underestimate costs and subsequently devalue the economic benefit of testing by ES. Ultimately, the difference in approaches needs to be further explored to determine which approach yields a truer cost and valuation of ES.

Differences in the accuracy of the final diagnosis between the unblind and blind experts may be explained by the type of diseases presented in each case. As Case 2 is difficult to diagnose without the use of ES, it is expected that the unblind group had a greater average diagnostic accuracy and less change in diagnosis across visits. Prior knowledge of the test result likely resulted in false high accuracy of clinical predictions due to overconfidence in the counterfactual scenario in the unblind group. This observation is also supported by the creeping determinism hypothesis, a type of hindsight bias. This hypothesis predicts that in retrospect, individuals who know an outcome will perceive this outcome to have a higher probability of occurring than individuals who do not know an outcome (19). It follows that unblind experts would assign greater confidence scores to the correct diagnosis than blind experts.

We do not see effects of creeping determinism or overconfidence, if present, in Cases 1 and 3, because the unblind experts had no advantage from knowing the ES result for these disease types. The Case 1 patient was undiagnosed by ES, so the unblind experts do not have a definitive, singular outcome that can bias their judgments. The unblind experts had to predict diagnoses with a similar level of knowledge as the blind experts. The Case 3 patient had a molecular diagnosis of pyridoxine-dependent epilepsy, which can be easily diagnosed without ES, and all experts correctly diagnosed this patient.

The use of the unblind approach in estimating the counterfactual scenario may fail to demonstrate the true value of ES for clinical outcomes, especially in Case-2-type patients, by indicating that there is little difference in diagnostic accuracy between patients who receive ES and those who do not. This would result in subsequent underestimation of the cost effectiveness of ES.

Given that only patients with Case 2 disease types may benefit the most from ES, as they are difficult to diagnose without ES, the relative prevalence of each disease type may also affect the overall value of ES for intractable pediatric epilepsy patients. In our 104-patient cohort, Case 2 represents the most common disease type, accounting for 60.5 percent of the cohort, followed by Case 1 (35.5 percent) and Case 3 (3.8 percent) (21). Although this cohort is of small size, other similar cohorts also report a significant number of cases that would benefit from ES, for example, patients with an unknown etiology of epilepsy. Of 114 infantile epilepsy cases in Australia, 33 percent had unknown causes (24), and similarly, of 663 pediatric epilepsy cases in Jordan, 40 percent had unknown causes (25). Given the relative numbers of patients who would likely benefit the most from ES, both disease type and prevalence should be taken into consideration when performing an economic evaluation of this intervention.

Further analysis of the value for money of ES is forthcoming. A similar study comparing cost and health outcomes from expert judgment between groups, one group with the option to perform rapid ES or rapid genome sequencing and one group without the option, will provide additional insight into both the clinical and economic benefit of ES.

Given the lack of significance, any observed differences in prognostic accuracy between the unblind and blind groups could have resulted by chance, or due to a too small sample size. Psychological effects may not have been evident in this outcome, as a patient’s prognosis cannot be definitively determined from ES results. For this judgment task, unblind experts are likely less influenced by hindsight bias because they do not know the prognosis of each patient.

Prognostic accuracy, similar to diagnostic accuracy, is also likely related to disease type. Diseases that are more well understood or have definitive treatment plans may yield greater prognostic accuracy. For example, vitamin B6 supplementation can treat seizures in patients with pyridoxine-dependent epilepsy (Case 3), and we see high confidence for the correct prognosis, no seizures, in both unblind and blind experts for this case (Supplementary Table S3).

Comparison of the unblind and blind approach to expert judgment in our study did not show conclusive evidence that there is a difference. However, we suspect that the outcomes from the blind approach are truer given that this approach more accurately simulates the counterfactual scenario. Experts are asked to provide judgments assuming that they did not know the ES result, as captured by the unblind group, is less representative of the counterfactual scenario. Even if experts are aware that knowledge of clinical outcomes may affect their judgments, previous studies have suggested that individuals are unable to ignore or adjust to these effects (23). Further research is needed to understand which, if either, approach is more accurate, and how this may impact decision-making.

To the best of our knowledge, this is the first simulation study exploring the potential benefits of using the blind approach in estimating costs and clinical outcomes of the counterfactual scenario of a personalized medicine intervention, for which there is limited evidence available from randomized control trials or other research methods. We believe our findings on the use of the unblind versus blind approach for the evaluation of ES are applicable to other types of precision medicine as well. Until further evidence suggests otherwise, we recommend the blind approach as preferable for expert judgment evaluation of other precision medicine technologies for which the counterfactual scenario has to be considered as a comparison, for example, when real-world data are limited. The use of the blind approach may require a bit of additional time and cost to organize compared to the unblind approach. For example, patient cases and experts may need to be recruited from separate hospitals to ensure blinding. However, additional effort and cost are minimal and would be beneficial given that expert judgment outcomes are often used to inform decision-making.

There are several limitations to this study, including the sampling size and sampling method. The small sample of both experts and case studies likely resulted in a lack of significance in the results for direct medical costs and accuracy of predicted prognosis. Although the Reference Case recommends a minimum of five experts for a judgment task to be sufficient (20), a sample size calculation may be more appropriate to consider for this type of study. However, as our judgment task occurred in person during the COVID-19 pandemic, this posed significant barriers to expert recruitment.

Another consideration is that the counterfactual scenario may be oversimplified for the purpose of the judgment exercise. In reality, clinicians may have more, or different, investigation and treatment options than was presented in each case study. Likewise, patients likely have a greater frequency of visits in reality, which could result in unreliable cost and health outcomes from the study. Although it may be impossible to simulate a counterfactual scenario that perfectly represents reality, efforts to reduce discrepancies should be taken to provide more accurate estimations.

This type of study is crucial to understand how precision medicines can be accurately evaluated for their economic and clinical benefit, and further research is needed in this area. Given the potential impact of these results, we recommend that similar studies be conducted in alternative settings and with an evaluation of other types of precision medicine technologies. Increasing the sample size of both experts and case studies in the future may result in more conclusive outcomes.

## Supporting information

Dulsamphan et al. supplementary material Dulsamphan et al. supplementary material

## Supplementary material

The supplementary material for this article can be found at https://doi.org/10.1017/S0266462323002714.
