# Supplementary material for: Can knowledgeable experts assess costs and outcomes as if they were ignorant? An experiment within precision medicine evaluation
Source: Int J Technol Assess Health Care. 2023 Nov 17;40(1):e4. doi: 10.1017/S0266462323002714 (PMC10859837; doi:10.1017/S0266462323002714)
Supplement: Dulsamphan et al. supplementary material [file S0266462323002714sup001.docx]

**SUPPLEMENTARY MATERIALS**

**Table S1. Expert Characteristics**

|  | **Total** | **Unblind** | **Blind** |
| --- | --- | --- | --- |
|  | **N** | **N (%)** | **N (%)** |
| **Gender** | | | |
| Female | 9 | 4 (57.1) | 5 (71.4) |
| Male | 5 | 3 (42.9) | 2 (28.6) |
| **Affiliation Hospital** | | | |
| Public | 12 | 6 (85.7) | 6 (85.7) |
| Private | 2 | 1 (14.3) | 1 (14.3) |
| **Province** | | | |
| Bangkok | 11 | 6 (85.7) | 5 (71.4) |
| Other | 3 | 1 (14.3) | 2 (28.6) |
|  | | | |
| **Working experience (Years)** | **Total** | **Unblind** | **Blind** |
| Minimum | 1 | 1 | 1 |
| Maximum | 27 | 27 | 20 |
| Average | 8.3 | 9.6 | 7.0 |

**Table S2. Comparison of total percent change in diagnosis across visits between groups by t-test**

| **Case** | **Group**  **(N=14)** | **Mean (SD)** | **95% CI** |
| --- | --- | --- | --- |
| **1** | **unblind** | 114.3 (91.4) | 29.7, 198.9 |
|  | **blind** | 141.4 (80.5) | 67.0, 215.9 |
| **2** | **unblind** | 107.1 (37.7) | 72.3, 142.0 |
|  | **blind** | 194.3 (106.8) | 95.6, 293.0 |
| **3** | **unblind** | 156.9 (61.2) | 100.2, 213.5 |
|  | **blind** | 95.7 (96.6) | 6.4, 185.0 |

**Table S3. Descriptive findings of predicted prognosis by group**

| **Case** | **Group** | **Predicted prognosis (%)** | | | |
| --- | --- | --- | --- | --- | --- |
|  |  | **More frequent seizures** | **Same frequency of seizures** | **Less frequent seizures** | **No seizures** |
| **1** | **unblind** | 51^c^ | 36 | 11 | 1 |
|  | **blind** | 46^c^ | 43 | 11 | 0 |
| **2** | **unblind** | 26 | 47 | 22^c^ | 5 |
|  | **blind** | 42 | 39 | 19^c^ | 1 |
| **3** | **unblind** | 1 | 1 | 13 | 86^c^ |
|  | **blind** | 4 | 0 | 1 | 94^c^ |

^c^ indicates the correct prognosis
